# Thermal Transfer Characteristics of Flat Plate Micro Heat Pipe with Copper Spiral Woven Mesh and a Copper Foam Composite Wick

**DOI:** 10.3390/nano11112821

**Published:** 2021-10-24

**Authors:** Yanhui Zhang, Zhengang Zhao, Chuan Luo, Dacheng Zhang

**Affiliations:** 1 Faculty of Information Engineering and Automation, Kunming University of Science and Technology, Kunming 650500, China; 18863669939@163.com (Y.Z.); luochuan@kust.edu.cn (C.L.); dacheng.zhang@kust.edu.cn (D.Z.); 2 Yunnan Key Laboratory of Computer Technology Applications, Kunming 650500, China

**Keywords:** flat-plate micro heat pipe, composite wick, wettability modification, thermal efficiency

## Abstract

The thermal efficiency limitation of the Flat-plate Micro Heat Pipe (FMHP) is a major challenge in the development of the FMHP, where the effect of wick structure and wettability on its thermal performance is studied to improve the thermal efficiency of the FMHP. In this work, a copper spiral woven mesh and copper foam Composite Wick FMHP (CW-FMHP) is designed based on the conventional Copper Foam Wick FMHP (CFW-FMHP), and its thermal performance is analyzed regarding the wick structure and internal gas–liquid two-phase flow characteristics. An oxidized copper spiral woven mesh and copper foam Composite Wick FMHP (OCW-FMHP) has been further developed through the modification of composite wick wettability. The performance tests are carried out with the thermal transfer characteristics of CW-FMHP, OCW-FMHP, and CFW-FMHP under different filling rates and different thermal powers. The experimental results show that the thermal transfer performance of CW-FMHP reaches the optimal under a liquid filling rate of 150%, where the maximum thermal power is 15.7 W, 35.3% higher than that of the CFW-FMHP under the same filling rate. Moreover, the dynamic response characteristics of the CW-FMHP are significantly improved. The thermal resistance of the CW-FMHP is 0.48 °C/W under the filling rate of 150% at the thermal power of 10 W with a reduction of 9.4% compared to the CFW-FMHP under the same condition. Furthermore, the optimal filling rate for OCW-FMHP is lower compared with the CW-FMHP. The maximum thermal power of OCW-FMHP increases to 17.8 W while the thermal resistance reduces to 0.34 °C/W under the liquid filling rate of 140%. This implies that the composite wick structure designed in this work can improve the thermal transfer performance of the FMHP, and the composite wick with wettability modification is more effective regarding both thermal resistance and maximum thermal power.

## 1. Introduction

With the development of microelectronics, electronic chips become increasingly integrated while the heat generated per unit volume continues to ascend [1,2,3,4]. The lack of timely heat dissipation has become a major problem affecting the efficiency and service life of the chip. To install thermal dissipation devices in a small space requires thermal dissipation components with a small form factor, efficient thermal dissipation capability, and fast dynamic response. FMHP efficiently transfers heat from high power-consuming components, providing a passive, reliable, lightweight, and low-cost path for thermal dissipation in electronic devices such as mobile phones, computers and tablets, etc. [5,6,7,8]. Therefore, the preparation of FMHP by using the phase change latent heat technique has become a popular topic in the study of heat dissipation of microelectronic devices. Hence, it is of great importance to study miniaturization, high thermal dissipation efficiency, and fast dynamic response of FMHP [9,10,11,12].

The wick is one of the most important parts that determine the thermal performance in FMHP. The sintering process is applied to prepare various types of porous wick, and the thermal properties of heat pipes of different porosity, pore size, and permeability can be investigated. It has been proved that porous wick heat pipes have higher thermal transfer limits [13,14,15,16]. In 2016, Mizuta et al. [17] developed a flat vapor chamber with a fine mesh wick of 50 mm × 50 mm × 2 mm and found that this vapor chamber delivered a small thermal resistance. Zhou et al. [18] proposed a small toroidal heat pipe that used an internal wick structure made of sintered fine copper mesh, with a thermal resistance of 0.111 ${}^{{}^{\circ}}$C/W and an evaporator temperature of 97.2 ${}^{{}^{\circ}}$C at 11 W in gravity-favorable operation mode. In 2019, Li et al. [19] developed two kinds of thin vapor chamber, copper foam vapor chamber and copper mesh vapor chamber, to improve the thermal performance of cooling electronic equipment. The experimental results shown that the thermal performance of the copper foam vapor chamber was about 2 times higher than that of the copper mesh vapor chamber at 50 ${}^{{}^{\circ}}$C and 30 ${}^{{}^{\circ}}$C cooling water temperature. Liu et al. [20] studied the flat ring heat pipe with thin polyacrylonitrile carbon fiber wick. The chemical plating process was applied to coat the surface of the carbon fiber with copper so that the total thermal resistance of the heat pipe varied from 1.1 to 0.45 ${}^{{}^{\circ}}$C/W in the steady operation. Li et al. [21] used copper mesh wick to prepare FMHP. However, researchers [22,23,24] found that a single porosity wick could not maintain high permeability and strong capillary force at the same time, whereas the composite wick could achieve this goal and further improved the thermal performance of FMHP. In 2016, Xu et al. [25] introduced an ultra-thin FMHP where they bonded copper micropillars to a single layer of stainless steel mesh via electroplating to form a composite wick structure and demonstrated that the FMHP could operate within a heating area of 8 mm × 8 mm at a heat load of 7.9 W. In 2019, Huang et al. [26] developed a new stainless steel fiber-powder composite wick to obtain a permeability wick with higher capillary force than other single wicks. Zhou et al. [27] developed a new dual-porous spiral woven mesh wick to improve the thermal performance of FMHP for cooling high heat flux electronic devices, where the liquid flow characteristics were theoretically analyzed. They stated that the dual-porous wick has the advantages of large pores with high permeability and small pores with strong capillary force. Meanwhile, the dual-porous wick consumed 22% less material than the single-porous wick, which could meet the requirement of low production cost and high thermal performance. In 2020, Jafari et al. [28] investigated an FMHP with a multi-scale feature wick that showed better thermal performance at high heat fluxes compared to conventional wicks. Researchers [29,30,31,32] also found that the maximum thermal power of FMHP depended on the dryness status of the evaporator section. Furthermore, chemical corrosion could modify the wick wettability and accelerate the liquid return rate to alleviate the dryness. In 2020, Chen et al. [33] found that the internal surface treatment could significantly improve the thermal performance of microgroove aluminum flat heat pipe, where the optimal treatment time was 10 min with the solution concentration of 1.5 mol/L. The thermal performance increased approximately 80% while the thermal resistance had been reduced by over 44% compared to untreated microgroove aluminum flat heat pipes. In 2021, Tang et al. [34] proposed a high-performance FMHP with a woven structure prepared by oxidation treatment and sintering. They found that the oxidation treatment efficiently improved the thermal transfer capacity of FMHP. Those studies showed that the FMHP of the porous material wick was the development trend, and surface wettability modification was an effective way to improve FMHP thermal properties.

In this study, an FMHP with a composite wick is proposed by considering the structural characteristics of FMHP with the porous material wick to improve its thermal dissipation efficiency. The thermal performance of FMHP is analyzed by studying the effects of wick structure, vapor flow channel volume, and wettability on the gas–liquid two-phase flow characteristics. Three types of FMHP (CW-FMHP, CFW-FMHP, and OCW-FMHP) are prepared. Their performances are tested under different liquid filling rates and different thermal power conditions. Through a comparative study of their maximum thermal power, the optimal liquid filling rate of CW-FMHP is determined, and the dynamic response characteristics and thermal resistances are analyzed under the optimal filling rate.

## 2. CW-FMHP Structural Design and Preparation

The analysis of the influence on the wick structure, the vapor flow channel volume, and the wettability on the gas–liquid two-phase flow characteristics can provide theoretical support for the structural design of the CW-FMHP. Then, the heat transfer power of CW-FMHP could be optimized based on the analysis of the gas–liquid two-phase flow characteristics.

### 2.1. CW-FMHP Structure

**Figure 1:**
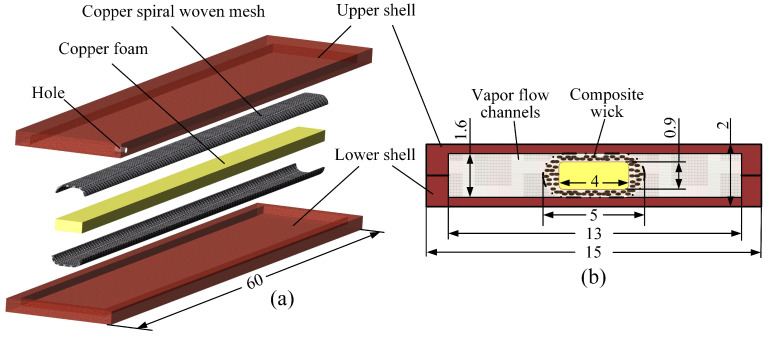
Schematic diagram of CW-FMHP measured in mm. (**a**) Exploded Views of CW-FMHP; (**b**) Cross section diagram of CW-FMHP.

The structure of CW-FMHP developed in this work is demonstrated in Figure 1. The CW-FMHP is encapsulated by a welded shell with a liquid injection path. Internal composite wick consists of the copper foam and a wrapped copper spiral woven mesh. It is pressed into the center of the CW-FMHP as a support to prevent shell deformation. When the evaporator section is heated, the liquid evaporates and passes through the vapor flow channels. Condensate is formed when steam is exothermic at the cooling end. The wick then transfers the condensate to the evaporator section and the gas–liquid two-phase cycle is formed.

### 2.2. Two-Phase Flow Characteristics

#### 2.2.1. Pressure Balance

The gas–liquid two-phase flow is the essential function of CW-FMHP, where the wick is the very part of enabling the function. The wick sends the liquid from the condenser section to the evaporator section, so that the condensed liquid can participate in the two-phase flow circulation. When CW-FMHP operates at low thermal power, the pressure drop at the two-phase interface caused by evaporation and condensation can be neglected. The temperature difference between the evaporating end and the cooling end is small, and the buoyancy provided by the temperature difference can be ignored. When the pipe is tested in the horizontal position, the flow of the liquid mainly depends on the capillary force provided by copper foam, and the reverse friction area between liquid flow and steam flow is very small. Thus, a simplified pressure model [21] can be used for analysis:

(1) $$\Delta P_{w,max}\geq \Delta P_{l}+\Delta P_{v}$$

where $\Delta P_{w,max}$ is the wick capillary force, $\Delta P_{l}$ is the flow pressure drop of liquid, and $\Delta P_{v}$ is the flow pressure drop of vapor. Increasing the capillary force of the wick or reducing the pressure loss caused by the flow of liquid or vapor can speed up the two-phase flow circulation. Neglecting the interaction at the two-phase flow interface, the flow pressure drop of vapor is given by:

(2) $$\Delta P_{v}=\frac{32\mu_{v}L_{eff}Q}{d_{v}^{2}A_{v}\rho_{v}h_{vl}}$$

where $\mu_{v}$ is the vapor viscosity, $L_{eff}$ is the effective length, *Q* is the thermal power, $d_{v}$ is the equivalent width of the vapor flow channels, $A_{v}$ is the cross-sectional area of the vapor flow channels, $\rho_{v}$ is the vapor density, and $h_{vl}$ is the latent heat of evaporation. It can be seen from Equation (2) that the flow pressure drop of vapor is inversely proportional to the cross-sectional area and the equivalent width, and is positively proportional to the thermal power. It implies that a clear airflow path can speed up the two-phase flow circulation within the CW-FMHP.

The flow-pressure drop of the liquid is related to the porosity, pore shape, pore size, and pore distribution in the direction of flow. It can be calculated by Darcy’s law [19]:

(3) $$\Delta P_{l}=\frac{\mu_{l}L_{w}m^{\prime }}{K\rho_{l}A_{w}}$$

where $\Delta P_{l}$ is the pressure drop of the liquid flow, $\mu_{l}$ is the viscosity of the liquid, $L_{w}$ is the wick length, K is the wick permeability, $m^{\prime }$ is the reflux mass of the liquid, $\rho_{l}$ is the density of the liquid, and $A_{w}$ is the wick cross-sectional area. The composite wick in this study is of multi-layer structure, in which more liquid flow channels are formed between the copper foam and copper spiral woven mesh sandwich. It helps to increase the wick permeability and reduce the pressure drop of the liquid flow [19,27]. Thus, the return flow of liquid from the condenser section to the evaporator section can be speeded up.

According to the Yang–Laplace formula [35], $\Delta P_{w,max}$ can be expressed by:

(4) $$\Delta P_{w,max}=\frac{2\sigma \cos\theta }{r_{eff}}$$

where σ is the surface tension of the liquid, θ is the contact angle of the material surface, and $r_{eff}$ is the effective capillary radius of the wick. The radius of the composite wick in this study is large, which results in a weak capillary force. According to Equation (4), the capillary force of the wick can be enhanced by reducing the contact angle of the material surface, and the circulation of the gas–liquid two-phase flow can be thus promoted.

#### 2.2.2. Composite Wick Wettability

Then, the wettability of the composite wick and its improvement will be investigated by using the Casey–Baxter and Wenzel models.

**Figure 2:**
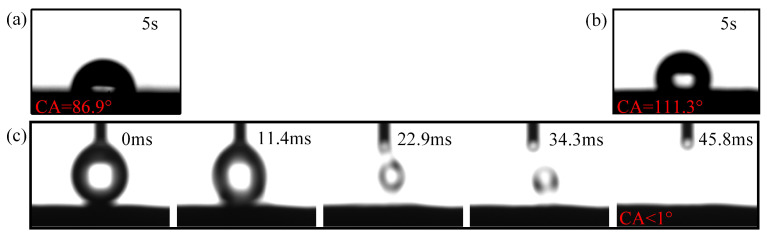
Contact angle measurement using 5 μL of deionised water. (**a**) Copper plate; (**b**) Porous material; (**c**) Composite wick after wettability modification.

When a water droplet touches a flat copper plate, the contact angle is $86.9^{{}^{\circ}}$, as shown in Figure 2a. When the water droplet comes into contact with the porous material with gas inside, the droplet could be suspended and the contact angle is $111.3^{{}^{\circ}}$, as shown in Figure 2b. The trapped gas under the water droplet reduces the material surface energy, which leads to a Casey–Baxter state. The correlation of the contact angle, the trapped gas under, and the the roughness of the infiltration surfaces are explained by the Casey–Baxter model [36]:

(5) $$\cos\theta_{CB}=R_{AF}F_{1}\cos\theta +F_{1}-1$$

where $\theta_{CB}$ is the Casey–Baxter contact angle, $R_{AF}$ is the roughness of the wetting area, and $F_{1}$ is the relative proportion of the solid surface projected by the liquid. Equation (5) shows that the more trapped gas under the water droplet, the smaller the relative proportion of the solid surface projected by the liquid, and the larger the Casey–Baxter contact angle. The small pores of internal copper foam can absorb liquid and the large pores of external copper spiral woven mesh can exhaust gas. During the operation, the external structure of the composite wick can exhaust in time, which can greatly reduce the internal vapor volume in the evaporator section. It helps to reduce the Casey–Baxter contact angle, which in turn enhances the capillary force of the composite wick and leverage the circulation of the gas–liquid two-phase flow.

In this study, the wettability modification process for the composite wick is described as follows:

1. The composite wick was ultrasonically cleaned in methanol and ethanol solution in turn for 10 min to remove the oil from the surface. Any subsequent operations were carried out with clean tweezers to prevent oil contamination;
2. The composite wick was ultrasonically cleaned with 1 mol/L HCl solution in a constant temperature chamber at 50 ${}^{{}^{\circ}}$C for 5 min and rinsed clean with deionized water;
3. The composite wick was then sealed in 125 mL of 30 %wt H${}_{2}$O${}_{2}$ and ultrasonicated for 15 min, placed in a constant temperature chamber at 100 ${}^{{}^{\circ}}$C for 3 h;
4. It was finally rinsed with deionized water and dried in a drying chamber at 70 ${}^{{}^{\circ}}$C.

**Figure 3:**
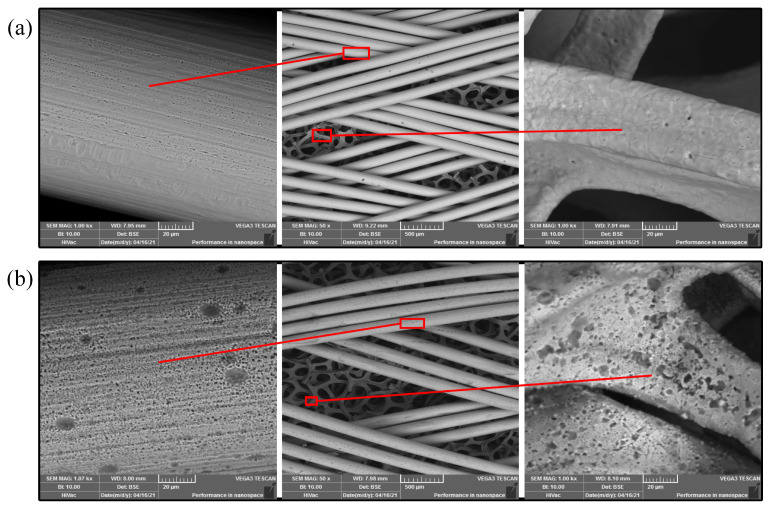
SEM image of composite wick. (**a**) Untreated composite wick; (**b**) Composite wick after wettability modification.

The Scanning Electron Microscope (SEM) images of the composite wick before and after the wettability modification is shown in Figure 3. Compared to the untreated composite wick, the surface of the composite wick after the wettability modification erodes a layer of the nanostructure. The infiltration behavior of the modified composite wick is shown in Figure 2c, where the water droplet is completely absorbed in about 45.8 ms, which is a very fast absorption process. When the liquid is completely infiltrated, when $F_{1}=1$, Equation (5) thus becomes the Wenzel model:

(6) $$\cos\theta_{w}=R_{AF}\cos\theta$$

where $\theta_{w}$ is the Wenzel contact angle. According to Wenzel’s theory, the chemical corrosion of a layer of nanostructure on the composite wick could increase its surface roughness, modify the surface wettability. Therefore the composite wick is made into a superhydrophobic structure, which enhances its capillary force, and the circulation of gas–liquid two-phase flow could be promoted.

### 2.3. Heat Transfer Power Analysis

The theoretical thermal transfer power can be calculated by:

(7) $$Q=m^{\prime }h_{vl}$$

where *Q* is the thermal power and $h_{vl}$ is the latent heat of evaporation. When the return mass $m^{\prime }$ of the composite wick reaches its maximum, $m^{\prime }$ = $m_{max}$, it gives:

(8) $$Q_{max}=m_{max}h_{vl}$$

where $Q_{max}$ is the maximum thermal power and $m_{max}$ is the maximum reflux mass, which is related to the velocity of the entire two-phase flow cycle.

In summary, the CW-FMHP developed in this study has a multi-layer structured wick inside with more fluid flow channels and higher permeability compared to the conventional CFW-FMHP. The composite wick absorbs liquid through internal small pores of copper foam, and exhausting gas through external large pores of copper spiral woven mesh, such that the impact of untimely vapor exhaustion at the evaporator section under high power can be diminished. Thus, a higher maximum thermal power can be achieved. Moreover, the chemical corrosion methods can modify the wettability of the composite wick and enhance its capillary force, which also contribute to the maximum thermal power.

### 2.4. CW-FMHP Preparation

**Figure 4:**
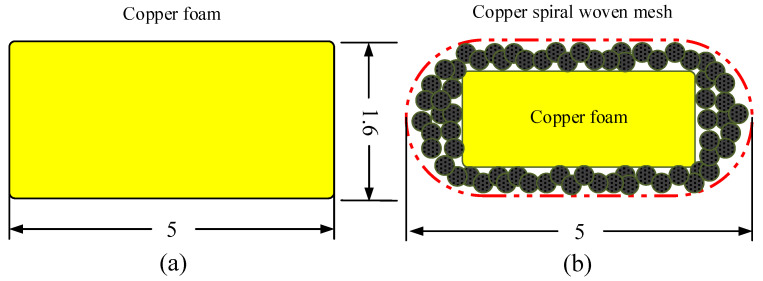
Schematic diagram of the cross-section of the wick in different configurations in mm. (**a**) Copper foam wick; (**b**) Copper spiral woven mesh and copper foam composite wick.

In this study, three types of FMHP are prepared: conventional Copper Foam Wick FMHP (CFW-FMHP), copper spiral woven mesh and copper foam Composite Wick FMHP (CW-FMHP), and Oxidized copper spiral woven mesh and copper foam Composite Wick FMHP (OCW-FMHP). A cross section view of the CFW and the CW is shown in Figure 4a and Figure 4b, respectively. The selected copper foam is 130 Pores Per Inch (PPI) and the selected copper spiral woven mesh has a copper wire diameter of 125μm. A large number of tiny liquid flow channels are formed between the copper spiral woven mesh and the copper foam sandwich, which facilitates faster liquid flow back from the condenser section to the evaporator section.

**Figure 5:**
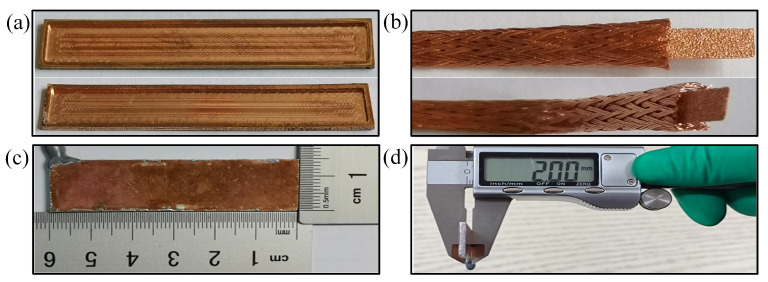
Shell and the composite wick. (**a**) Upper and lower shell; (**b**) Composite wick; (**c**) FMHP length and width; (**d**) FMHP thickness.

On both the upper and lower shells of the FMHP, microgrooves with the depth of 0.8 mm are milled, with a welding strip of 1 mm width. The side of the upper shell is drilled with a 0.5 mm diameter hole, as shown in Figure 5a. Then the internal walls of the upper and lower shells are polished smooth. A uniform layer of solder is applied to the solder strips of the upper and lower shells, and the wick is placed in the middle of the lower shell and soldered on a heated plate at 300 ${}^{{}^{\circ}}$C. Vacuum is drawn through a small hole reserved on the upper shell and liquid methanol is injected. The prepared FMHP is shown in Figure 5. The preparation process is manipulated within 12h to prevent volatile organic compounds from adhering to the wick and affecting its hydrophilicity [36]. Plus, the preparation process also prevents high-temperature sintering from destroying the nanostructure of the wettability-modified composite wick [21].

As shown in Figure 5b, the inner pore volume of the composite wick is not structurally equal to the sum of the inner pore volumes of the two materials. The porosity of the wick is measured by using the water saturation method [37]. The dried wick is first weighed using an electronic balance with accuracy of 0.1mg. Then, the wick is placed in deionized water, sonicated for 10 min before the saturated sample is weighed. To minimize the measurement error, the process is repeated three times and the average value is taken. The porosity is calculated by:

(9) $$\xi =\frac{V_{p}}{V_{w}}$$

(10) $$V_{p}=V_{sw}=\frac{M_{2}-M_{1}}{\rho }$$

where ξ is the wick porosity, $V_{p}$ is the total volume of pores, $V_{w}$ is the total volume of the wick, $V_{sw}$ is the volume of saturated water in the wick, $M_{1}$ is the weight of the wick in dry state, $M_{2}$ is its weight after water absorption and saturation, and ρ is the density of the deionized water. The results show that the porosity of the composite wick is 67.21% and the porosity of the copper foam wick is 70.24%.

Moreover, the filling rate could directly affect the fluidity of the vapor flow channels and the maximum thermal power of the FMHP. The liquid filling rate is calculated as:

(11) $$\mu =\frac{V_{l}}{V_{p}}\times 100\%$$

where μ is the liquid filling rate of the FMHP, $V_{l}$ is the volume of the filled liquid and $V_{p}$ is the total volume of the internal pores of the wick.

## 3. CW-FMHP Performance Test

Through the analyzing the internal gas–liquid two-phase flow characteristics of CW-FMHP, the influence of wettability on the thermal transfer power can be obtained. The design scheme of the wettability-modified composite wick is proposed. To verify the rationality of the theoretical analysis, three wick types of FMHP are prepared. A test platform is built to test their thermal performance.

### 3.1. Experiment Settings

**Figure 6:**
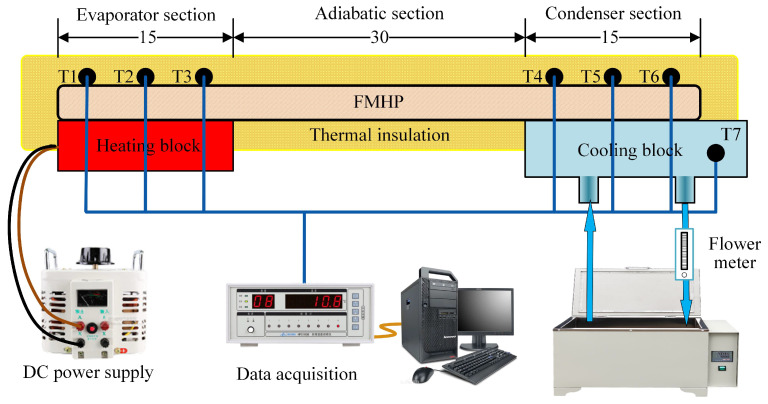
Diagram of the measurement system.

The test platform consists of a heating unit, a cooling unit, and a data acquisition unit, as shown in Figure 6.

**Figure 7:**
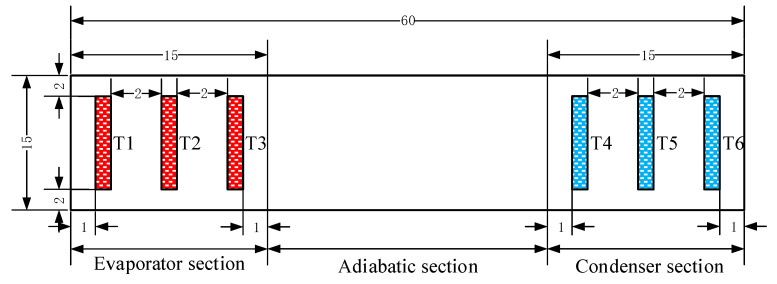
Diagram of the distribution of FMHP surface temperature collection points measured in mm.

The heating unit consists of a heating block and a DC power supply (Model UTP3313TFL-II) to provide thermal power input. The heating block uses a ceramic resistance with dimensions of 15 mm × 15 mm × 2 mm. The cooling unit consists of a cooling block, a water pump, a flow meter, and a thermostatic water bath. The cooling block is a copper block with a cavity inside, where the circulating water flows through. The contact area between the cooling block and the FMHP is 15 mm × 15 mm to ensure that the evaporator section and the condenser section have the same size. The cooling water flow rate is set to 250 L/h and the constant temperature water bath is set to 25 ${}^{{}^{\circ}}$C. The data acquisition unit consists of a temperature patrol detector (Model KCM-XJ16WRS), a PC terminal, and seven Pt100 Resistance Temperature Detectors (RTDs), which are numbered from T1 to T7. T1 to T6 are used to collect the axial temperature distribution of the FMHP, while T7 is used for the water temperature of cooling water. The diameters of Pt100 RTDs are 3 mm. The implementing positions of the thermal sensors are shown in Figure 7.

T1, T2 and T3 are evenly distributed at the evaporator section of the FMHP to collect the temperature at the evaporator section. T4, T5 and T6 are evenly distributed at the condenser section to collect the temperature at the condenser section. The gaps between the heating block, the cooling block, the Pt100 RTDs, and the FMHP are coated with a layer of thermally conductive silicone grease with thermal conductivity of 2.5 W/MK to reduce the contact thermal resistance. The entire test section is covered with asbestos with thermal conductivity less than 0.15 W/MK such that heat loss can be minimized.

During the test, the starting power is set to 1 W with the increment of 3 W [35]. When the surface temperature of the FMHP have stabilized around the cooling water temperature, the DC power supply is switched on to input thermal power. The temperature patrol meter collects data from the Pt100 RTDs in real-time. When the temperature of the six measuring points is stabilized within ±0.1${}^{{}^{\circ}}$C for the 60 s, the FMHP is considered to reaching a operating equilibrium. Increase the power in sequence until the maximum thermal power. The temperature data collected by the temperature patrol meter is transferred to the PC and recorded. The maximum thermal power is defined as the maximum load at which the FMHP can operate normally. The temperature difference between the evaporator section and condenser section is within 6 ${}^{{}^{\circ}}$C. The temperature indexes at the evaporator section and the condenser section is defined as the relative averages calculated by:

(12) $$T_{e}=\frac{T_{1}+T_{2}+T_{3}}{3}$$

(13) $$T_{c}=\frac{T_{4}+T_{5}+T_{6}}{3}$$

where $T_{e}$ and $T_{c}$ is the average temperatures at the evaporator section and the condenser section, respectively. The temperature difference is expressed as:

(14) $$\Delta T=T_{e}-T_{c}=\frac{\left(T_{1}+T_{2}+T_{3}\right)-\left(T_{4}+T_{5}+T_{6}\right)}{3}$$

The thermal resistance $R_{ec}$ of FMHP is defined as:

(15) $$R_{ec}=\frac{\Delta T}{Q}$$

where *Q* is the thermal power of the heating block. The measurement uncertainty mainly comes from the heating unit and the data acquisition unit. The maximum uncertainty of the heating unit is related to the DC power supply. The accuracy of the voltage and current of the DC power supply is 0.5%. The resolution of the temperature patrol meter in the data acquisition unit is 0.1${}^{{}^{\circ}}$C, and the maximum measurement error of the Pt100 RTDs is 0.03${}^{{}^{\circ}}$C. The uncertainties are calculated by:

(16) $$\frac{e(y)}{y}=\frac{\sqrt{\sum {\left(\frac{\partial y}{\partial x_{i}}ex_{i}\right)}^{2}}}{y}$$

where e(y) is the measurement error of *y*, which is a given function of $x_{i}$. $ex_{i}$ is the maximum measurement error of each variable $x_{i}$. The maximum uncertainties are 0.3% for the thermal power, 1.77% for the temperature difference, and 1.9% for the thermal resistance.

### 3.2. CW-FMHP Axial Temperature Distribution and Maximum Thermal Power

The thermal performance of CW-FMHP are compared with CFW-FMHP and OCW-FMHP regading the axial temperature distribution and the maximum thermal power.

#### 3.2.1. CW-FMHP Maximum Thermal Power under Different Filling Rate

**Figure 8:**
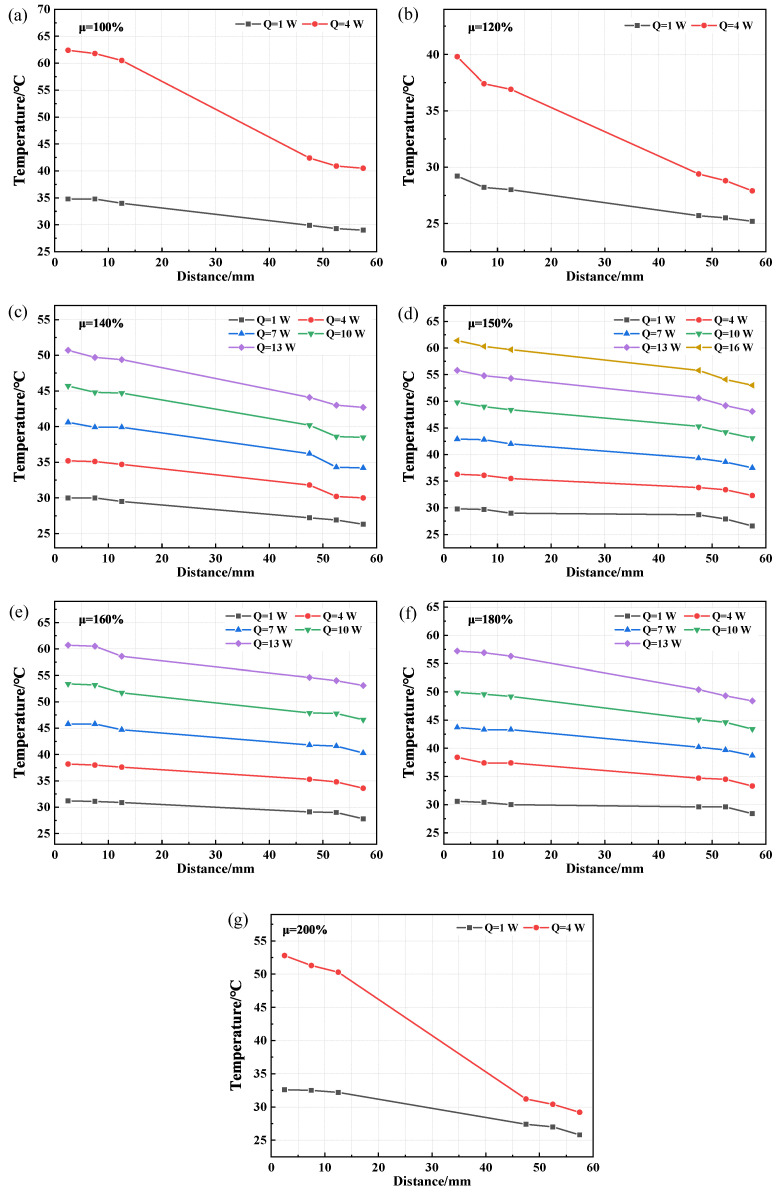
CW-FMHP axial temperature distribution at different thermal powers. (**a**) 100% filling rate; (**b**) 120% filling rate; (**c**) 140% filling rate; (**d**) 150% filling rate; (**e**) 160% filling rate; (**f**) 180% filling rate; (**g**) 200% filling rate.

**Figure 9:**
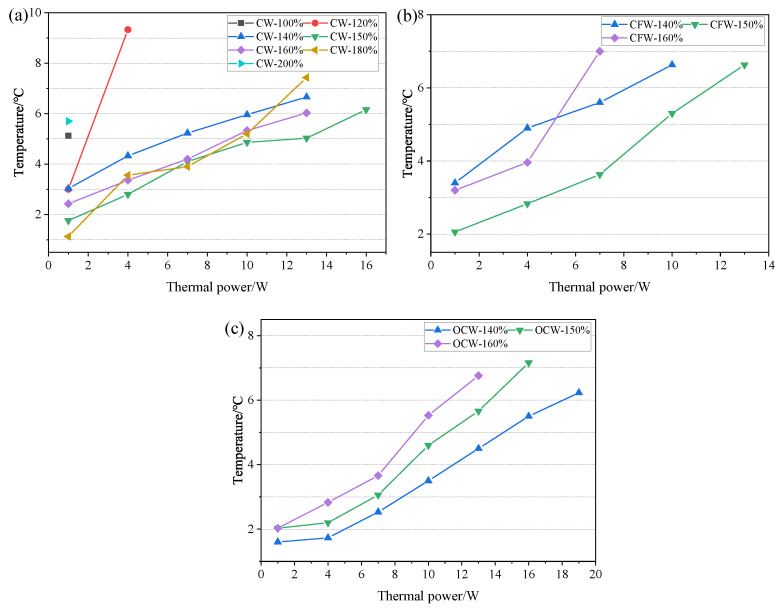
Temperature difference of FMHP under different thermal power. (**a**) CW-FMHP; (**b**) CFW-FMHP; (**c**) OCW-FMHP.

**Figure 10:**
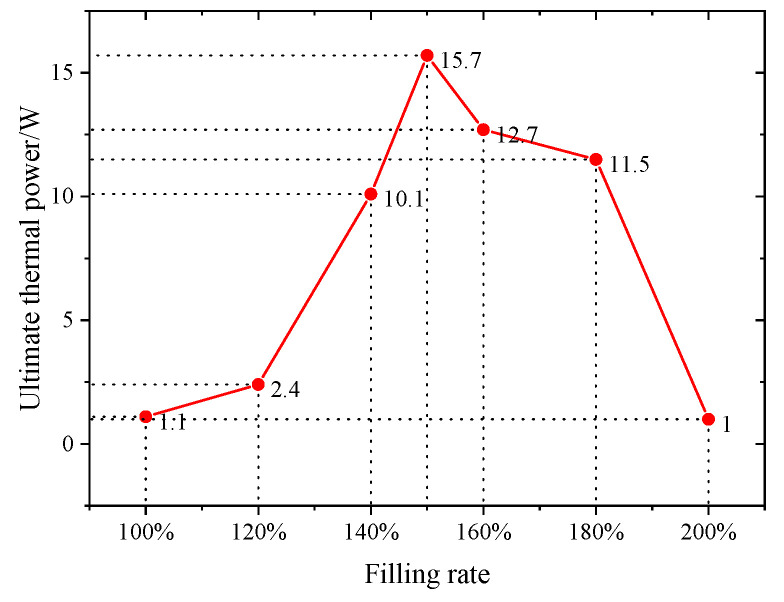
Maximum thermal power of CW-FMHP at different filling rates.

The axial temperature distribution and temperature difference of CW-FMHP under different thermal powers are shown in Figure 8 and Figure 9a. Furthermore, its maximum thermal power under different liquid filling rates are shown in Figure 10.

Under the filling rate of 100%, the temperature difference reaches 5.1${}^{{}^{\circ}}$C at the thermal power of 1 W, and the maximum thermal power of CW-FMHP gradually increases with the increasing filling rate. When the filling rate reaches 150%, the temperature difference is 5.0${}^{{}^{\circ}}$C at a thermal power of 13 W, with maximum thermal power of 15.7 W. As the filling rate continues to increase, the maximum thermal power of CW-FMHP starts to decrease. When the filling rate reaches 200%, the temperature difference is 5.7${}^{{}^{\circ}}$C at the thermal powerof 1 W. This is mainly because that, at low filling rates, more and more liquid is involved in the two-phase circulation as the thermal power continues to increase. When $m^{\prime }$ reaches the maximum return mass $m_{max}$, the evaporator section of the CW-FMHP starts to dry out, resulting in the remaining thermal energy not being removed by the two-phase flow, and the surface temperature of the CW-FMHP rises sharply and reaches the maximum thermal power. At this stage, the maximum thermal power of CW-FMHP can be further increased by accelerating the liquid filling rate such that the maximum reflux mass $m_{max}$ will be leveraged as well. Thus, the dryness phenomenon at the evaporator section will be alleviated, which contributes to the improvement of the maximum thermal power. When the capillary limit of the composite wick is reached, the excess liquid filling rate does not effectively participate in the two-phase cycle. Instead, it will block the vapor flow channels of the CW-FMHP, which leads to the resistance to vapor flow and maximum thermal power is thus reduced. It can be seen that the optimal filling rate of the CW-FMHP is around 150%.

The axial temperature distribution and the maximum thermal power of CW-FMHP under a range of 120–180% filling rates are studied. As shown in Figure 8 and Figure 9a, the temperature difference of CW-FMHP with 120% filling rate at the thermal power of 4 W is 9.3${}^{{}^{\circ}}$C. The temperature difference of CW-FMHP under 180% filling rate at the thermal power of 4 W is 3.5${}^{{}^{\circ}}$C, 5.9${}^{{}^{\circ}}$C under 140% filling rate at 10 W, and 5.3${}^{{}^{\circ}}$C under 160% filling rate at 10W. It can be seen that when CW-FMHP is tested at the same thermal power, the temperature difference under lower filling rates is higher than the the ones under higher filling rates. As shown in Figure 10, the maximum thermal power under 120%, 140%, 160%, and 180% filling rates are 2.4 W, 10.1 W, 12.7 W, and 11.5 W, respectively. It implies that the thermal performance of CW-FMHP under low filling rates deteriorates more significantly. The impact of drying out at the evaporator section under low filling rates is severer than the impact of excess liquid blocking the vapor flow channels at high filling rates. This is due to the excess filling rate under high filling rates increases the maximum reflux mass $m_{max}$, which contributes partly to the reflux of the liquid.

#### 3.2.2. Comparation of CW-FMHP, CFW-FMHP and OCW-FMHP

**Figure 11:**
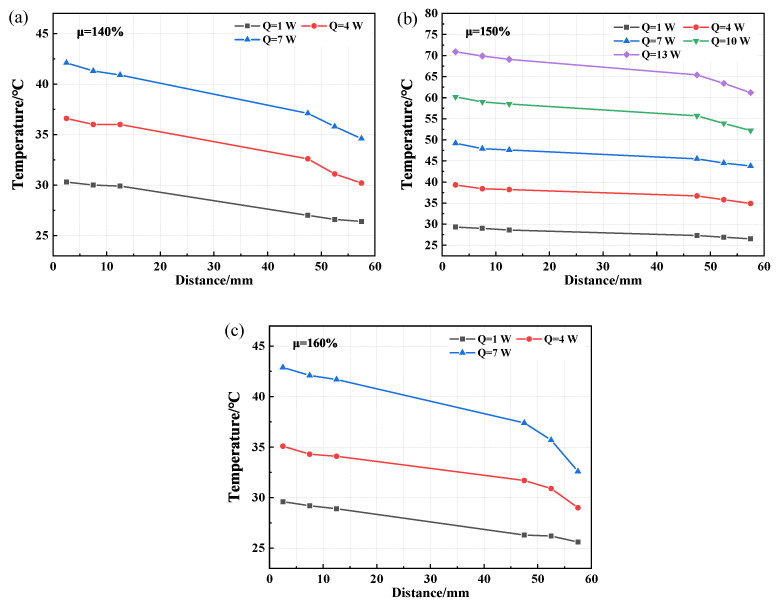
Axial temperature distribution of CFW-FMHP under different thermal power. (**a**) 140% filling rate; (**b**) 150% filling rate; (**c**) 160% filling rate.

**Figure 12:**
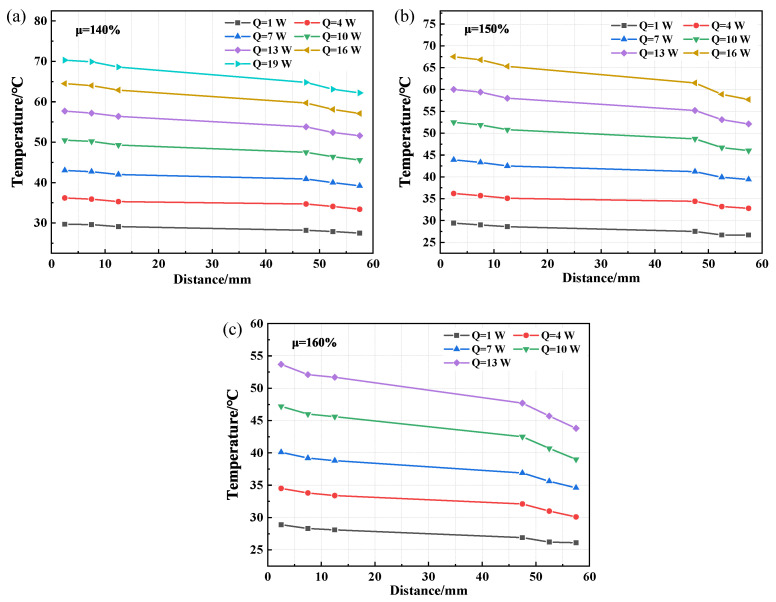
Axial temperature distribution of OCW-FMHP under different thermal power. (**a**) 140% filling rate; (**b**) 150% filling rate; (**c**) 160% filling rate.

Since the optimal filling rate for the CW-FMHP is around 150%, three filling rates of 140%, 150%, and 160% are chosen for the comparison to study the wick structures. The axial temperature distributions of CFW-FMHP and OCW-FMHP under different filling rates at different thermal powers are shown in Figure 11 and Figure 12. Their maximum thermal power values are listed in Table 1.

The temperature difference of CFW-FMHP is 5.3${}^{{}^{\circ}}$C under the filling rate of 150% at the thermal power of 10 W. The maximum thermal power decreases when it is under a filling rate either greater or smaller than 150%, as shown in Figure 9b and Figure 11. It can be deemed that the optimal liquid filling rate of CFW-FMHP is around 150%. Compared with CFW-FMHP, the maximum thermal power of the CW-FMHP is increased by 35.3% (4.1 W), as shown in Table 1. From the wick structure point of view, the number of pores in the copper foam wick is larger than that in the composite wick, thus the capillary force of the copper foam is greater than that of the composite wick. However, due to the large number of small pores in the copper foam, it is difficult for the vapor generated in the evaporator section to diffuse out of the copper foam wick. The increase of the Casey–Baxter contact angle of the copper foam wick could weaken the its capillary force, which in turn reduces the maximum reflux mass $m_{max}$. On the other hand, the vapor in the evaporator section is easily discharged through the large pores in the copper spiral woven mesh and barely impacts the contact angle of the composite wick. Therefore, the maximum thermal power of CW-FMHP is higher than that of CFW-FMHP.

Compared with CW-FMHP, the maximum thermal power of OCW-FMHP is increased by 2.1 W (Table 1). When it is under the filling rate of 140% at the thermal power of 16 W, the temperature difference of the OCW-FMHP is 5.5${}^{{}^{\circ}}$C, as shown in Figure 9c. When the rate is greater than 140%, the maximum thermal power of OCW-FMHP gradually decreases (Figure 12). It can be seen that the optimal filling rate for OCW-FMHP moves along with a smaller filling rate. The reason is that after the composite wick wettability modification, the capillary force and the maximum reflux mass $m_{max}$ increase, which improves the maximum thermal power and eventually leads to an increase in the concentration of vapor. The extra filling rate has a stronger impact on vapor impedance than on liquid reflux promotion, thus the optimal filling rate of OCW-FMHP moves towards a lower filling rate.

To summarize, the temperature at the six points from T1 to T6 decreases sequentially from the evaporator section to the condenser section. The axial temperature of the FMHP gradually increases as the thermal power increases, and eventually reaches the maximum thermal power. When the liquid filling rate of the FMHP is not appropriate and the capillary force of the wick is weak, the temperature distribution in the evaporator and condenser sections will fluctuate. It is because that a smaller liquid flow rate or vapor flow rate can negatively impact the circulation of the gas–liquid two-phase flow, which results in a disordered flow of liquid/vapor inside the FMHP. The temperature fluctuations in the evaporator and condenser sections can be improved by appropriately selecting the filling rate and increasing the capillary force of the wick.

### 3.3. Dynamic Response Characteristics

**Figure 13:**
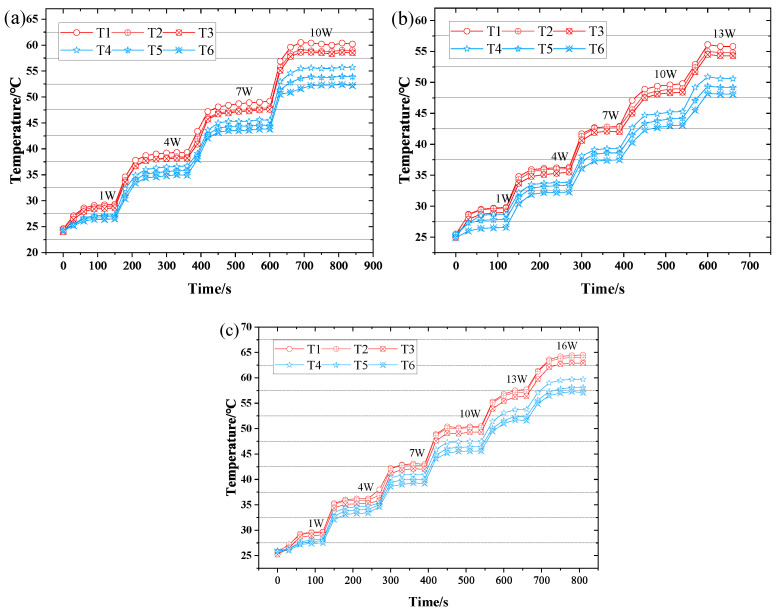
Dynamic characteristics of FMHP. (**a**) CFW-FMHP under 150% filling rate; (**b**) CW-FMHP under 150% filling rate; (**c**) OCW-FMHP under 140% filling rate.

Dynamic response characteristic is an important index of FMHP. The shorter the response time, the higher the heat transfer efficiency. This study investigates the performance of FMHPs of different structural types by comparing their dynamic response characteristics at different thermal powers. The dynamic response characteristics of CW-FMHP, CFW-FMHP, and OCW-FMHP under optimal liquid filling rate at increasing thermal power is shown in Figure 13. As the thermal power rises, the temperature at each measurement point on the FMHP surface can reach an equilibrium in a short period. The time required to reach the equilibrium at the surface temperatures of the CW-FMHP and OCW-FMHP are almost the same at the same thermal power level, which indicates that the FMHP with the composite wick structure has nearly the same dynamic response characteristics. Compared with CW-FMHP, the dynamic response characteristic of CFW-FMHP becomes worse with the increase of thermal power. The reason is that the vapor generated in the evaporator section is difficult to exhaust through the small pores in the copper foam. As the thermal power increases, the more vapor content inside the wick, the severer impact on the return speed of the liquid and the diffusion rate of the vapor. The vapor generated in the evaporator section of CW-FMHP and OCW-FMHP can be easily exhausted through the large pores in the copper spiral woven mesh and does not affect the gas–liquid circulation inside the FMHP.

### 3.4. Thermal Resistance

**Figure 14:**
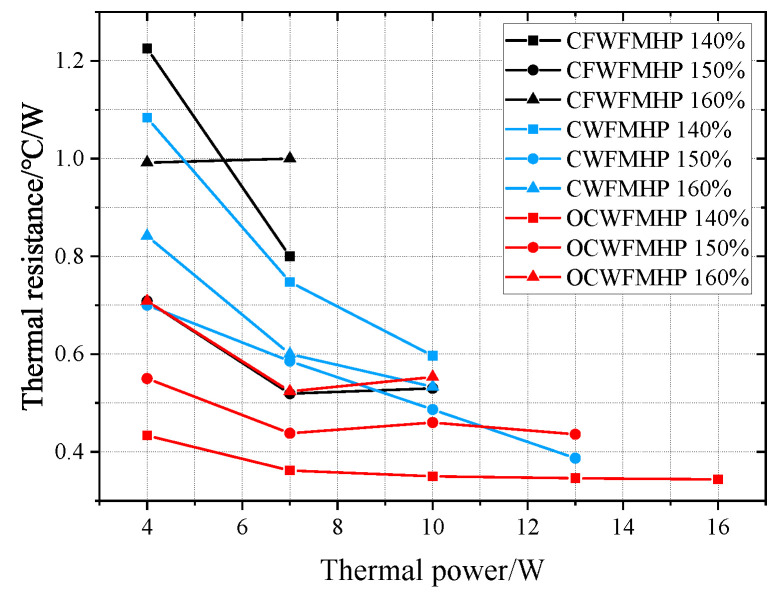
Thermal resistance of FMHP at different liquid filling rates.

The thermal resistance curves of CW-FMHP, CFW-FMHP, and OCW-FMHP under different filling rates are shown in Figure 14.

The thermal power is from 4 W to the maximum thermal power. As can be seen from Figure 14, the thermal resistance of the FMHP is larger at low thermal powers, and decreases when the thermal power increases. It is due to that the lower thermal power cannot fully vaporize the liquid in the evaporator section of FMHP. The less vapor cannot reach the condenser section smoothly, which increases the circulation resistance of the two-phase flow. Then the temperature difference and thermal resistance of FMHP are leveraged.

The total thermal resistance of the CFW-FMHP under the filling rate of 140% and 160% are larger than that of the CFW-FMHP under the filling rate of 150%. The performance of CFW-FMHP at 140% and 160% fill rate is poor. They are mainly because that under low filling rates, extra thermal energy cannot be carried away, and the temperature in the evaporator section significantly rises, which causes an increase in thermal resistance. The excess liquid under higher filling rates blocks the vapor flow channels and increases the flow resistance of the vapor, which also leads to an increase in thermal resistance. When the liquid filling rate is 150%, the thermal resistance of CW-FMHP is 0.38${}^{{}^{\circ}}$C/W at the thermal power of 13 W, and 0.48${}^{{}^{\circ}}$C/W at the thermal power of 10 W, which is 9.4% less than that of CFW-FMHP with 10 W thermal power. However, the thermal resistance of the CFW-FMHP is smaller than that of the CW-FMHP, at around 7 *W* thermal power. It is because that the capillary force of the copper foam wick is stronger than that of the composite wick, but the exhaust capacity is weaker. At lower thermal power, the evaporator section produces less vapor, and the capillary force of the wick itself dominates, whereas at higher thermal power, the evaporator section produces more vapor, and the exhaust capacity dominates.

The thermal resistance of OCW-FMHP can be reduced to 0.34${}^{{}^{\circ}}$C/W when under the liquid filling rate of 140% at the thermal power of 16 W. This is because that the capillary force of the composite wick becomes stronger after the wettability modification, and the liquid presents less resistance to reflux. However, comparing the total thermal resistance of CW-FMHP and OCW-FMHP under the filling rates of 150% and 160%, it can be seen that the thermal resistance of CW-FMHP is slightly lower than that of OCW-FMHP at the maximum thermal power under the same rate. This phenomenon is due to the weaker exhaust capacity of the composite wick after the wettability modification, which leads to an untimely exhaust at the maximum power that impacts the fluid return speed, which causes an increase in thermal resistance.

## 4. Conclusions

A flat-plate micro heat pipe with copper spiral woven mesh and copper foam composite wick structure is designed and studied in this work. Three kinds of FMHP with different wick types (CW-FMHP, OCW-FMHP, and CFW-FMHP) are prepared and used for comparative analysis. The optimal liquid filling rate of CW-FMHP is around 150%, and the maximum thermal power of CW-FMHP under 150% liquid filling rate is 15.7 W. The measured value of the thermal resistance is 0.38${}^{{}^{\circ}}$C/W at the thermal power of 13 W. The thermal performance of CW-FMHP under low filling rates can be significantly reduced compared with that under higher filling rates.

The composite wick structure allows for the absorption of liquid from the small internal pores and the exhaust of gas from large external pores. The higher the thermal power, the clearer the advantages of the composite structure are compared to the copper foam wick. Under a filling rate of 150%, the maximum thermal power of the CW-FMHP is 35.3% (4.1 W) higher than that of the CFW-FMHP, and the dynamic response characteristics are significantly improved. The thermal resistance of the CW-FMHP is 0.48${}^{{}^{\circ}}$C/W under the rate of 150% at the thermal power of 10 W, which is 9.4% (0.05${}^{{}^{\circ}}$C/W) smaller than taht of CFW-FMHP.

The use of chemical corrosion for the composite wick wettability modification can enhance its capillary force and thus improve the thermal performance. The maximum thermal power of OCW-FMHP can be promoted to 17.8 W and the thermal resistance can be reduced to 0.34${}^{{}^{\circ}}$C/W. The proposed method for wettability modification may reduce the exhaust capacity of the composite wick, but it can be negligible under an optimal liquid filling rate.

## Figures and Tables

**Table 1:** Maximum thermal power of FMHP with different wick types.

Wick Type	Filling Rate	Maximum Thermal Power
Copper Foam (CFW-FMHP)	150%	11.6 W
Composite (CW-FMHP)	150%	15.7 W
Oxidized Composite (OCW-FMHP)	140%	17.8 W

## Data Availability

Not applicable.

## Abbreviations

FMHP	Flat-plate Micro Heat Pipe
CW	Composite Wick
CFW	Copper Foam Wick
OCW	Oxidized Composite Wick
P	Pressure
L	Length
Q	Thermal power
A	Cross-sectional area
h${}_{vl}$	Latent heat of evaporation
μ	Viscosity;Liquid filling rate
ρ	Density
*K*	Permeability
$m^{\prime }$	Liquid reflux mass
σ	Surface tension
θ	Contact angle
*r*	Capillary radius
$R_{AF}$	Roughness
$F_{1}$	Relative proportion of liquid–solid contact
$m_{max}$	Maximum reflux mass
%wt	%Weight
*V*	Volume
ξ	Porosity
*M*	Mass
*T*	Temperature
ΔT	Temperature difference
$R_{ec}$	Thermal resistance
